# Stereotaxic Coordinates and Morphological Characterization of a Unique Nucleus (CSF-Contacting Nucleus) in Rat

**DOI:** 10.3389/fnana.2019.00047

**Published:** 2019-05-09

**Authors:** Si-Yuan Song, Yue-Hao Li, Cheng-Yi Bao, Ying Li, Peng-Cheng Yin, Jia Hong, Wan-Lin Li, Yuan Shi, Li-Cai Zhang

**Affiliations:** Jiangsu Province Key Laboratory of Anesthesiology, Xuzhou Medical University, Xuzhou, China

**Keywords:** CSF-contacting nucleus, stereotaxic coordinates, three-dimensional reconstruction, morphology, rat

## Abstract

A unique nucleus, the cerebrospinal fluid (CSF)-contacting nucleus, has recently been recognized in the brain parenchyma. The outstanding feature of this nucleus is that the neural somas are located in the parenchyma, but their processes stretch into the CSF, implying that it may be a key structure bridging the nervous and body fluids-regulating systems and may play a pivotal role in modulating physiological activities. However, the true biological significance of this nucleus needs to be uncovered. The morphology of a nucleus is one of the most important parameters for neuroscience studies. For this reason, a common experimental animal, Sprague-Dawley (SD) rats, was chosen. The position, adjacent structures, neuronal distribution, size, three-dimensional reconstruction, and core coordinates of the CSF-contacting nucleus in SD rats of different weights (90–400 g) were illustrated for the first time. Furthermore, the formulas for calculating the core coordinates of the CSF-contacting nucleus in rats of different weights were revealed. Finally, the possible biological functions uncovered by past research are reviewed in this paper. This study provides an indispensable methodology and a significant reference for researchers interested in this unique nucleus.

## Introduction

The transmission of signals between the nervous tissue and body fluids, despite the barriers in the brain, has remained a mystery (Liddelow, 2011). Although there are still some controversial viewpoints about the integrity of these barriers, the differing components of the extracellular fluid in the brain parenchyma and those of the cerebrospinal fluid (CSF) and blood prove that the nervous tissue and the CSF and blood in the brain are separated from one another (Rambeck et al., 2006). Maintaining a constant inner-environment is crucial (Ransohoff and Engelhardt, 2012); however, signal transmission between the nervous tissue and the CSF and blood exists objectively in the brain.

Although the brain has conventionally been regarded as lacking a classical lymphatic drainage system, Louveau et al. (2015) have recently reported that there are lymphatic vessels in the central nervous system (CNS). This discovery may call for a reassessment of the basic assumptions in neuroimmunology. Accordingly, are there any neural structures mediating the signal transmission between the neurons and the fluids in the brain?

Over decades, with the advance of tract-tracing techniques, other researchers have reported neurons or their processes that lie in the CSF of the lateral ventricle (LV), third ventricle (3V), and fourth ventricle (4V) and the central canal (CC) of the spinal cord in different species (Korf and Fahrenkrug, 1984; Nagatsu et al., 1988; Hirunagi et al., 1992; David et al., 2003; Stoeckel et al., 2003; Xiao et al., 2005). Vigh and Vigh-Teichmann (1998) and Vigh et al. (2004) regard the CSF-contacting neurons (CSF-CNs) as a peculiar cell type of the CNS and suppose that they play a role in non-synaptic signal transmission in the brain.

The functions of the CSF-CNs were recently revealed by novel methods (Jay and McDearmid, 2015; Petracca et al., 2016; Orts-Del’Immagine and Wyart, 2017; Orts-Del’Immagine et al., 2017). However, reviewing these studies of the CSF-CNs allows for the conclusion that they are scattered neurons, but do not form a nucleus, which are mainly located within structures near the ventricular system, such as ependymal and subependymal layers, or the ventricle cavity (Vigh et al., 2004; Calle et al., 2005; Xiao et al., 2005; Sato et al., 2011). Few studies have reported CSF-CNs located within the brain parenchyma far from the ventricular system because distinguishing these cells from non-CSF-CNs without a specific labeling method is difficult. Some studies have tried to inject horseradish peroxidase (HRP) or autoradiography substances into the ventricular system to label these neurons; however, these substances are likely to freely pass through the CSF-brain barrier, thereby labeling both the CSF-CNs and non-CSF-CNs. Therefore, the presence of these CSF-CNs is difficult to conclude scientifically.

The identification of a labeling reagent (or method) that labels only CSF-CNs, but not non-CSF-CNs, is therefore key for identification of the CSF-CNs within the brain parenchyma.

In 1992, we injected cholera toxin B subunit (CB)-HRP, a peripheral nervous system tracer, into the LV and found that it was only confined to the ventricular system but was unable to freely pass through the CSF-brain barrier into the brain parenchyma. Intriguingly, there is a specific brain region that consistently contains numerous CB-HRP-labeled neurons (Wang and Zhang, 1992; Zhang et al., 1994). The specific labeling pattern of CB-HRP suggests that only neurons that have processes that extend into the CSF are labeled. For more than 20 years, our studies have sufficiently confirmed that these CSF-CNs, located within this particular brain region, not only have synaptic and non-synaptic morphological connections with other functional structures (non-CSF-CNs, blood vessels, glia cells, and CSF) in the brain, but also have ultra-structures that mediate substance exchange, information transmission and functional modulation between the brain and body fluids (Zhang et al., 2003; Liang et al., 2007). Because these neurons exist in a consistent location, form an independent cluster, and are composed of the same type of neurons, which are entirely different from other already known nuclei in the brain, we therefore name it the “cerebrospinal fluid-contacting nucleus” or the “CSF-contacting nucleus” (Lu et al., 2011; Liu H. et al., 2014).

The concept of the CSF-contacting nucleus may bring a new “element” to brain structures, as well as be the “source” location at which substances in the CSF are exchanged under certain physiological and pathological situations. In addition, the presence of the CSF-contacting nucleus may explain the CNS effects of drugs (such as lumbar anesthesia) and implanted cells (such as stem cells and chromaffin cells) delivered into the CSF. Moreover, the unique features of the morphological connections of the CSF-contacting nucleus imply that it participates in both neuron–neuron and neuron–body fluids crosstalk and may play a significant role in both the neural and fluid adjustment mechanisms that affect all physiological processes. We firmly believe that identifying and understanding this nucleus will open up broad research fields and will attract more researchers to study this unique nucleus.

The morphology of a nucleus is one of the most important parameters for its neurobiological study. For this reason, we have accumulated data from the previous 20 years to repeatedly confirm and verify the CSF-contacting nucleus. We have then further clarified its location, neuronal distribution, three-dimensional spatial profile and stereotaxic coordinates from the surface of the skull. To easily identify its location, we have provided the core location coordinates of the CSF-contacting nucleus depending on weight. This study will provide necessary methodological support for researchers who are interested in this nucleus.

## Materials and Methods

### Experimental Animals

Specific pathogen-free (SPF) grade Sprague-Dawley (SD) rats, weighing 90–400 g, were acquired from the Experimental Animal Center of Xuzhou Medical University, license number SCXK (Jiangsu) 2015-0009. All experiments were approved and performed in accordance with the Committee for Ethical Use of Laboratory Animals, Xuzhou Medical University.

### Tracer Administration

Rats were anesthetized with pentobarbital sodium (40 mg/kg, i.p.), and heads were fixed on the stereotaxic instrument (Stoelting, United States). Then, 3 μl 30% CB-HRP (Sigma, United States), a specific tracer of the CSF-contacting nucleus by way of the ventricular system, was injected into the LV according to the stereotaxic coordinates provided by Paxinos and Watson (2007). After 48 h, rats were perfused and sacrificed.

### Rules of Sampling and Sectioning

The three points of the head, the incisor and bilateral external acoustic pores, were fixed on the stereotaxic instrument (Stoelting, United States). The incisor bar was set at approximately −3.9 mm to ensure that Bregma and Lambda were at the same coronal level, which is the standard position for sampling and sectioning. The brain segments were isolated in reference to Bregma, which was the landmark used on the skull surface. The coronal sections were sectioned from rostral to caudal, the sagittal sections were collected laterally from the midline, and the horizontal from dorsal (superficial) to ventral (profundal). The coronal and sagittal sections were cut at right angles to the horizontal plane joining Bregma and Lambda. All sections were cut at a consistent 40-μm thickness, kept in sequence from the beginning to the end, and numbered.

### Tracer Staining, Positive Neurons Quantification, and Neurotransmitter Confirmation

All sections received CB-HRP immunofluorescent staining (rabbit anti-CB primary antibody diluted in 1:600, Abcam; donkey anti-rabbit Alexa Fluor 488 secondary antibody diluted in 1:200, Life Technologies). Then, the sections were mounted in sequence on the slides, counterstained by DAPI, and coverslipped. The sections of different positions were captured at uniform parameter using a fluorescence microscope (Leica DM6, Germany). The number of positive neurons was counted by the Image-Pro Plus 7.0 software. Part of the sections received CB-HRP/serotonin immunofluorescent double labeling to confirm the neurotransmitter (rabbit anti-CB primary antibody diluted in 1:600, Abcam; goat anti-serotonin primary antibody diluted in 1:600, Abcam; donkey anti-rabbit Alexa Fluor 488 secondary antibody diluted in 1:200, Life Technologies; donkey anti-goat Alexa Fluor 594 secondary antibody diluted in 1:200, Life Technologies).

### The Stereotaxic Coordinates of the Nucleus

The stereotaxic reference system was based on the flat skull position, in which Bregma and Lambda lay in the same coronal level. This same position was the standard position used for sampling and sectioning. Bregma on the skull surface was the zero-reference point. The beginning and end points of the CSF-contacting nucleus were determined by the first and last section that had positive neurons in the serial sections of the three planes. The coronal plane was perpendicular to the rostral-caudal line passing through Bregma; the sagittal plane was perpendicular to the middle-lateral line passing through Bregma; the horizontal plane was perpendicular to the dorsal-ventral line passing through Bregma. The length unit of the coordinates is micron (μm). The coordinates of a random section on the coronal axis (C), sagittal axis (S), and horizontal axis (H) were the distances of that section to the zero-reference point (Bregma), namely, the section thickness (40-μm) multiplied by the sequence number of the section.

### The Reconstruction of the Nucleus in the Brain

The pictures of the serial sections with CB-HRP-positive labeling were automatically captured by the microscope (Leica DM6, Germany). The three-dimensional morphology of the CSF-contacting nucleus in the brain was reconstructed with Imaris software version 8.4.1 (Bitplane, United States).

### Calculating the Core Coordinates of the Nucleus in Rats of Different Weights

Animals were divided into three groups: low (L, approximately 100 g), middle (M, approximately 200 g), and high (H, approximately 300 g) weight depending on body weight. The tracer administration, the rules of sampling and sectioning, the tracer staining, the quantification of positive neurons and the identification of the stereotaxic coordinates of the nucleus were similar to the methods described above. The core of the CSF-contacting nucleus was determined by the section that had the most positive neurons.

### Statistics

SPSS 13.0 software was used for data analysis in the present study. Data were presented as the mean ± SD.

## Results

### The Labeling Characteristics of the Tracer in the Brain

**FIGURE 1:**
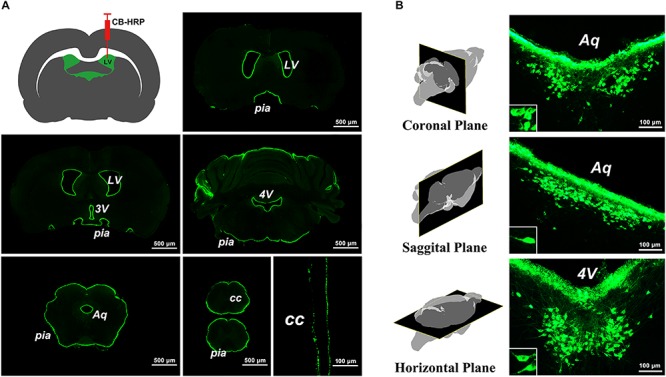
The labeling characteristics of the tracer CB-HRP in the brain. **(A)** The green fluorescence-labeled tracer is confined to the ventricular system. Bar = 500 μm; last bar = 100 μm. **(B)** The neuron cluster labeled by the tracer is consistently found within the same region of the brain parenchyma in the coronal, sagittal, and horizontal planes. Bar = 100 μm. (LV, lateral ventricle; 3V, 3rd ventricle; Aq, aqueduct; 4V, 4th ventricle; CC, central canal; pia, pia mater).

The green fluorescence-labeled tracer was confined to the ventricular system and formed a clear outline of the LV, 3V, aqueduct (Aq), 4V, CC, and pia mater of the brain and spinal cord (Figure 1A). A positively labeled neuron cluster consistently located in the same brain region within coronal, sagittal, and horizontal planes was found in the serial sections. The somas of these neurons appear to be fusiform or polygonal in shape (Figure 1B). Because the positive neuron cluster was consistently located within the same region in the brain and was labeled by the specific tracer, CB-HRP, by the way of ventricular system, it is called the CSF-contacting nucleus.

### The Position and Adjacency of the CSF-Contacting Nucleus

**FIGURE 2:**
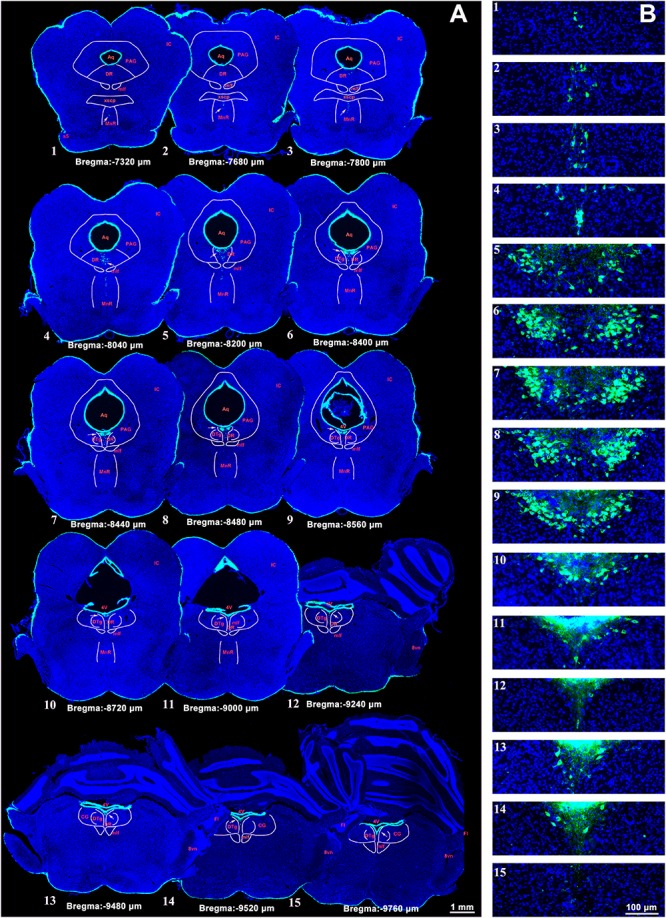
A systematic illustration of the location of the CSF-contacting nucleus in the brain. **(A)** The CSF-contacting nucleus (green) in the representative serial sections. Bar = 1 mm. **(B)** Higher magnification of the CSF-contacting nucleus in **(A)**. Bar = 100 μm. (IC, inferior colliculus; Aq, aqueduct; PAG, periaqueductal gray; DR, dorsal raphe nucleus; mlf, medial longitudinal fasciculus; xscp, decussation of the superior cerebellar peduncle; MnR, median raphe nucleus; s5, trigeminal nerve root; 4V, the 4th ventricle; DTg, dorsal tegmental nucleus; CG, central gray; 8vn, vestibular cochlear nerve root; Fl, cerebellum flocculus).

The CSF-contacting nucleus labeled by CB-HRP is consistently located within the ventral gray of the lower portion of the Aq and upper portion of the 4V floor. The rostral part of the nucleus begins in the plane formed by the superior border of the midbrain inferior colliculus (IC) and trigeminal nerve root (s5) at the middle pons. In this plane (Bregma −7,320 μm), the structures dorsal to the nucleus are the decussation of the superior cerebellar peduncle (xscp), median raphe nucleus (MnR), medial longitudinal fasciculus (mlf), periaqueductal gray (PAG), and dorsal raphe nucleus (DR), respectively. In this plane, the CSF-contacting nucleus is far from the Aq (Figure 2). Then, the nucleus extends dorsally and stretches into the ventral gray of the Aq. The core of the nucleus in the coronal section is symmetrical and appears in a Y-like shape (Bregma −8,480 μm). The nearby structures in this plane from ventral to dorsal are the mlf, DR, and dorsal tegmental nucleus (DTg) (Figure 2). The caudal part of the nucleus is located in the central gray (CG) in the upper portion of the 4V floor (Bregma −9,760 μm). The caudal border of the nucleus is located within the plane formed by the vestibular cochlear nerve root (8vn) and cerebellum flocculus (Fl) (Figure 2).

### Neurotransmitter Confirmation in the CSF-Contacting Nucleus

**FIGURE 3:**
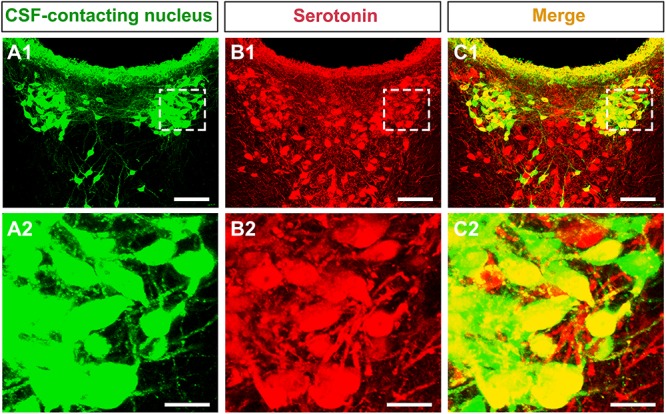
The neurotransmitter confirmation of the CSF-contacting nucleus in the brain. **(A1,B1,C1)** The CSF-contacting nucleus (green), serotonin (red), and double-labeling (yellow). Bar = 100 μm. **(A2,B2,C2)** Higher magnification of the boxed area in **(A1,B1,C1)**, respectively. Bar = 20 μm.

The CSF-contacting nucleus immunofluorescent double labeling reveals that most of the neurons in the nucleus (green) contain the serotonin, a neurotransmitter in the brain. Both of the neural somas and processes appear serotonin immuno-positive labeling (Figure 3).

### The Distribution of the Neurons in the CSF-Contacting Nucleus

**FIGURE 4:**
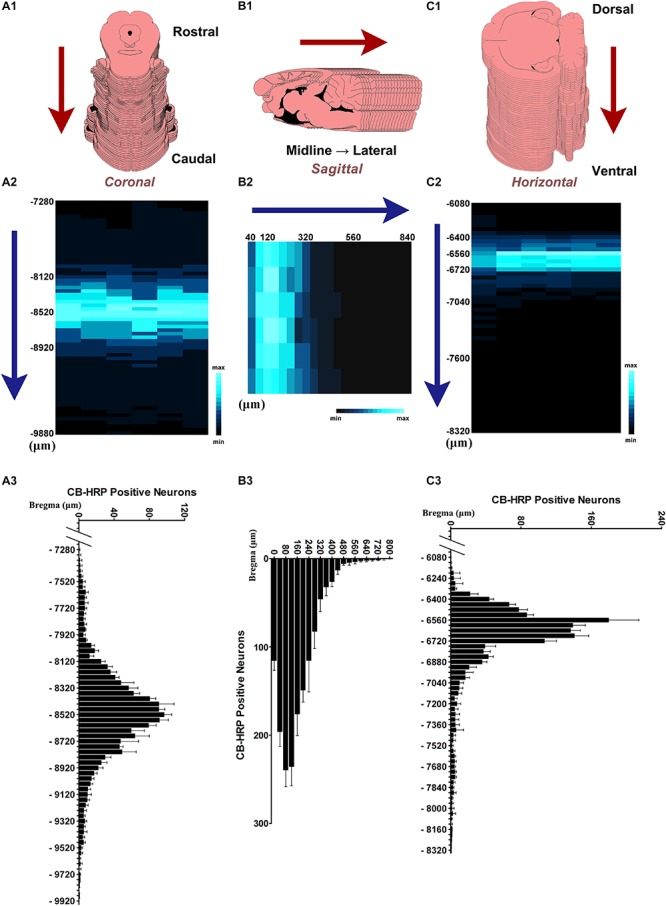
The distribution of the neurons in the CSF-contacting nucleus. **(A1,B1,C1)** Schematic diagram of the serial sections. **(A2,B2,C2)** Color map of neurons distribution in the CSF-contacting nucleus in coronal, sagittal, and horizontal planes, respectively. **(A3,B3,C3)** Neuron number distribution in the CSF-contacting nucleus in coronal, sagittal, and horizontal planes, respectively (*n* = 6). Data are expressed as mean ± SD.

In 300 ± 5 g SD rats (*n* = 6), the CSF-contacting nucleus began in the coronal plane at approximately 7,300 μm caudal to Bregma. Approximately 60 serial sections contained positive neurons. The number of neurons in each serial section increased gradually, until it reached the peak near Bregma −8,500 μm. In the core section of the CSF-contacting nucleus, there were as many as 102 ± 10 neurons, and the nucleus was symmetrical and appeared as a Y-like shape. The number of neurons then decreased gradually until they disappeared near Bregma −9,700 μm. The total length of the nucleus was approximately 2,400 μm (Figure 4A). In the sagittal plane (*n* = 6), Bregma is located just above the longitudinal cerebral fissure. Beginning in the brain midline, approximately 20 serial sections both to the left and to the right contained positive neurons. The number of neurons reached its peak with as many as 244 ± 16 neurons at 80–120 μm lateral to the midline. Then, the number of neurons decreased gradually until they disappeared. The maximum width of the nucleus reached approximately 800 μm (Figure 4B). In the horizontal plane (*n* = 6), the CSF-contacting nucleus began approximately 6,100 μm below the skull surface. There were approximately 50 serial sections in the horizontal plane that contained positive neurons. The number of neurons peaked at as many as 181 ± 31 neurons at approximately 6,600 μm below the skull surface. Then, the number of neurons decreased gradually until the disappeared at approximately 8,300 μm below the skull surface. The maximum depth of the nucleus was approximately 2,200 μm (Figure 4C).

### The Size, Core, and Three-Dimensional Reconstruction of the CSF-Contacting Nucleus in the Brain

The start and the end of the CSF-contacting nucleus were defined as the first and last section positions containing CB-HRP-positive neurons on the serial sections, and the core of the CSF-contacting nucleus was defined as the section position containing the most CB-HRP-positive neurons. The distance between the start and end of the nucleus in the coronal, sagittal, and horizontal plane are the length (L), width (W), and depth (D) of the nucleus, respectively, namely, the section thickness (40 μm) multiplied by the sequence number of the section from Bregma. The size (L, W, and D) and core of the nucleus in rats of a common experimental weight are shown in Table 1.

**TABLE 1:** The size and core of the CSF-contacting nucleus inrats of a common experimental weight (*n* = 6, $\bar{x}$ ± s).

Plane	Weight (g)	Origin (μm)	End (μm)	Size (μm)	Core(μm)
Coronal		7427 ± 109	9767 ± 64	2340 ± 90 (L)	8500 ± 33
Sagittal	300 ± 5	0	747 ± 33	747 ± 33 (W)	93 ± 21
Horizontal		6140 ± 42	8227 ± 70	2087 ± 109 (D)	6573 ± 33

**FIGURE 5:**
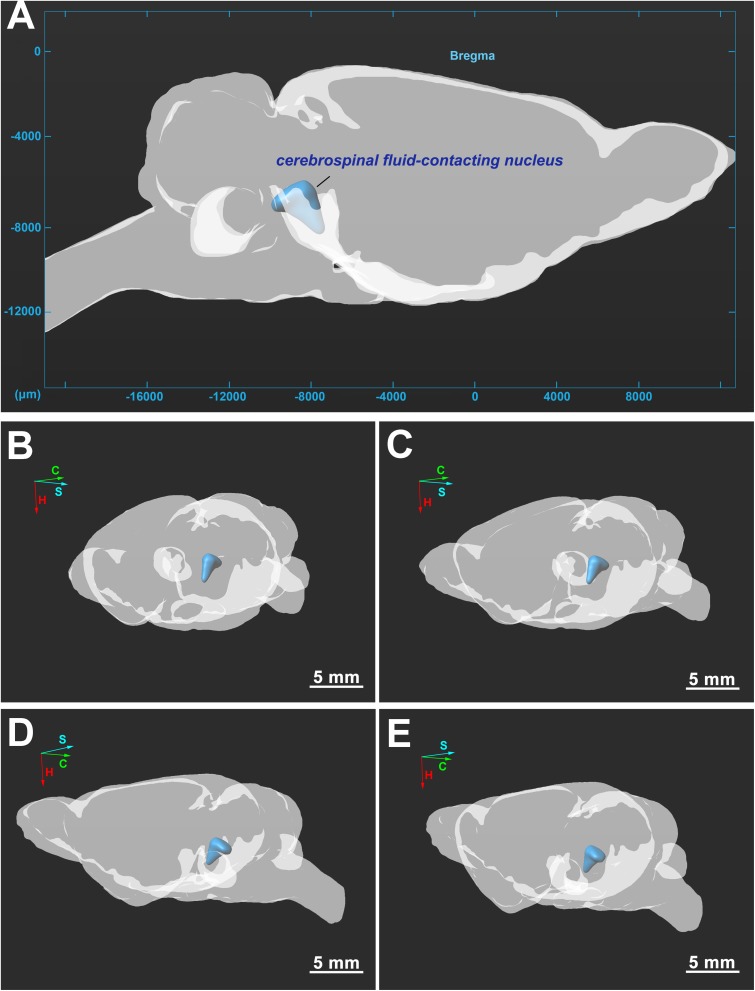
**(A–E)** Three-dimensional structure of the CSF-contacting nucleus in the brain from different perspectives. C, coronal; S, sagittal; H, horizontal. Bar = 5 mm in **(B–E)**.

The three-dimensional morphology of the CSF-contacting nucleus in the brain was reconstructed with Imaris software. The CSF-contacting nucleus presents a definite spatial morphology within the brain with clear spatial boundaries and location. The nucleus exists independently and consistently in the brain appearing as a rivet-like shape between the inferior segment of the midbrain to the superior segment of the pons. The head portion of the nucleus is wide, and the tail is thin (Figure 5).

### The Core Coordinates of the CSF-Contacting Nucleus in Rats of Different Weights

The core coordinates (coronal, sagittal, horizontal) of the CSF-contacting nucleus in different groups of rats according to a low (L), middle (M), or high (H) weight are shown in Table 2.

**TABLE 2:** The core coordinates (coronal, sagittal, horizontal) of the CSF-contacting nucleus in different weight groups(*n* = 6, $\bar{x}$ ± s).

Group	Weight (g)	Coronal (μm)	Sagittal (μm)	Horizontal (μm)
L	101 ± 6	7707 ± 33	73 ± 16	6100 ± 22
M	200 ± 5	8040 ± 36	87 ± 16	6393 ± 53
H	321 ± 8	8513 ± 30	100 ± 22	6580 ± 33

**FIGURE 6:**
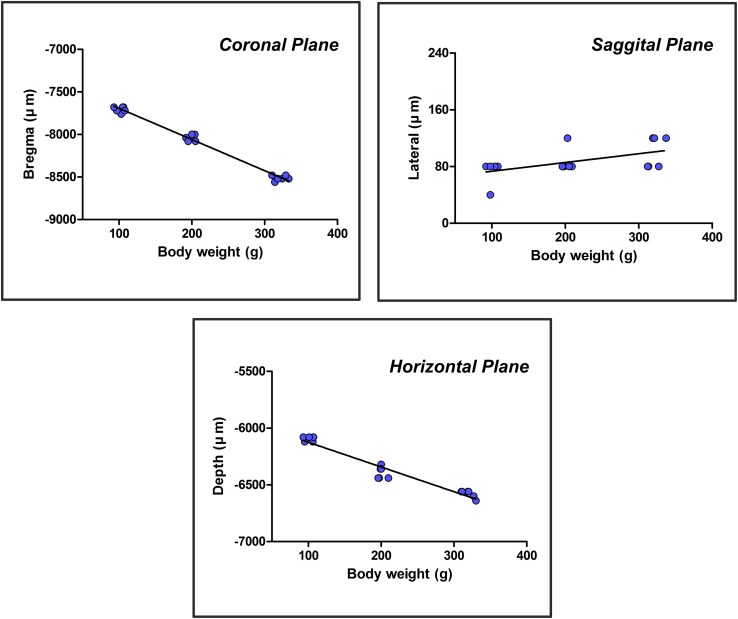
A scatter diagram and linear regression of the core three-dimensional coordinates of the CSF-contacting nucleus.

The independent variable, weight (*x*), was plotted on the horizontal axis, and the dependent variable, the core coordinate of the CSF-contacting nucleus (coronal, sagittal, and horizontal planes) (*Y*), was plotted on the vertical axis to establish the rectangular coordinate system. The above experimental data were used to create the scatter diagrams of the rectangular coordinate system. The relationship between the two variables was estimated by a linear regression in which “*a*” is the intercept, and “*b*” is the regression coefficient. Once “*a*” and “*b*” were determined, the core coordinates of the CSF-contacting nucleus (coronal, sagittal, and horizontal) (*Y*) in rats of different weights (*x*) could be calculated by the formula *Y* = *a*+*bx*. The scatter diagrams and linear regression of the core coordinates of the CSF-contacting nucleus in three groups of rats are shown in Figure 6.

The formulas were acquired based on the scatter diagram and linear regression. The core coordinates of the CSF-contacting nucleus correlate with body weight significantly (*F*_1,16_ = 1060.539, *P* < 0.001 for coronal; *F*_1,16_ = 7.376, *P* < 0.05 for sagittal; *F*_1,16_ = 262.243, *P* < 0.001 for horizontal). *R*-values are 0.993, 0.562, and 0.971 for coronal, sagittal, and horizontal planes. In a weight range of 90–400 g, the formulas are as follows: *Y*^Coronal^ = 7325.26 + 3.667*x* (μm); *Y*^Sagittal^ = 61.02 + 0.124*x* (μm); and *Y*^Horizontal^ = 5906.57 + 2.179*x* (μm). The “*x*” in the formulas is the body weight (g) of the rat.

## Discussion

### Further Confirmation of the CSF-Contacting Nucleus

Our previous studies have repeatedly proven that injection of the tracer CB-HRP into the LV of the rat forms a clear outline along the LV, 3V, Aq, 4V, CC of the spinal cord, and the surface of the brain and spinal cord. These results confirm that CB-HRP only runs within the ventricular system and does not freely pass through the CSF-brain barrier into the brain parenchyma. Evidently, the CB-HRP-positive neurons are CSF-CNs whose processes stretch into the ventricular system and directly contact the CSF. Their somas are consistently located within the same position in the brain, form a cluster, and are differently shaped than their nearby non-CSF-contacting structures in the brain parenchyma, which fit the definition of a nucleus in the brain (Moore et al., 2006; Blumenfeld, 2010; Purves, 2012). Therefore, we have termed this unique neuron cluster the “cerebrospinal fluid-contacting nucleus” or “CSF-contacting nucleus,” not to be confused with “CSF-CNs” or “CSF-contacting neuron.”

Notably, we once regarded this neuron cluster as a part of the DR or as distal CSF-CNs of the brain parenchyma in our early studies (Zhang et al., 2003; Liang et al., 2007). However, upon further investigation, an increasing number of studies have repeatedly confirmed that the CSF-contacting nucleus is entirely different from the DR. The DR, located ventral to the PAG, is shaped like a fountain and is also called the “spring nucleus” (Wang and Zhang, 1995; Paxinos and Watson, 2007; Liu Z. et al., 2014). The CSF-contacting nucleus begins ventral to the xscp, and the nearby structures from ventral to dorsal are the xscp, MnR, mlf, PAG, and DR, respectively. The main portion of the CSF-contacting nucleus in coronal sections is symmetrical and Y-shaped. The caudal part of the CSF-contacting nucleus stretches dorsally from the ventral gray of the PAG toward the CG of the upper floor of the 4V. The stereo spatial structure of the CSF-contacting nucleus in the brain was illustrated by using a three-dimensional reconstruction technique to demonstrate the morphology and spatial position of the nucleus more fully than with conventional section observations alone. The nucleus has a rivet-like shape and exists independently and consistently between the inferior segment of the midbrain and the superior segment of the pons. The CSF-contacting nucleus is an intrinsic and independent functional structure that is entirely different from the DR and other previously defined structures in the brain. The CSF-contacting nucleus has a unique spatial location, defined boundaries, and quantifiable size, as well as specific neural types and other defining features. Therefore, we have sufficient and reliable scientific evidence to identify CSF-contacting nucleus as a unique and independent nucleus in the brain.

### The Stereotaxic Coordinates of the CSF-Contacting Nucleus and Methodological Application

The stereotaxic coordinates of brain atlases have provided indispensable tools for neuroscience studies (Paxinos et al., 2000; Paxinos and Franklin, 2004; Paxinos and Watson, 2007; Lanciego and Vazquez, 2012). Previously, there were no precise stereotaxic coordinates describing the location of the CSF-contacting nucleus as a unique region in the brain.

We used the repeatedly confirmed tracer, CB-HRP to label the CSF-contacting nucleus. We have made serial coronal, sagittal, and horizontal sections at a constant 40 μm thickness and successfully determine the stereotaxic coordinates of the CSF-contacting nucleus. Moreover, the core stereotaxic coordinates of the CSF-contacting nucleus highly correlate with the body weight significantly. Therefore, the core stereotaxic coordinates can be estimated according to body weight.

When the experimental SD rats are between 90 and 400 g, import the weight “*x*” (g) into the formulas, *Y*^Coronal^ = 7325.26 + 3.667*x* (μm), *Y*^Sagittal^ = 61.02 + 0.124*x* (μm), and *Y*^Horizontal^ = 5906.57 + 2.179*x* (μm), to obtain the CSF-contacting nucleus core coordinates according to the linear equations. Under anesthesia, fix the head of the rat on a stereotaxic instrument using three points of the head, the incisor and bilateral external acoustic pores. Then, remove the scalp, and expose the skull surface. Set the incisor bar at approximately −3.9 mm to ensure that Bregma and Lambda are in the same coronal plane (the standard position). Move the scales of the stereotaxic instrument to the intersection of the *Y*- and *X*-axis coordinates. Then, remove the scales, and drill a hole into the skull at the specified location with a dental drill. Fix a microsyringe on the stereotaxic instrument at the calculated *Y*- and *X*-axis coordinates and lower the microsyringe to the depth identified by the *Z*-axis value. Thus, the intended reagent can be injected into the core of the CSF-contacting nucleus. The injection site can also be verified by observing the trace of the needle tract and the specific location of the needle tip. Because the core of the CSF-contacting nucleus is not a single point but rather an area, the probability of hitting the core of the CSF-contacting nucleus is relatively high.

### Initial Functional Implications of the CSF-Contacting Nucleus

After discovering that delivering CB-HRP via the brain ventricular system can specifically label the CSF-contacting nucleus, we have studied this unique nucleus for approximately 25 years (Wang and Zhang, 1992). Many biological functions of the CSF-contacting nucleus have been suggested by the results of multiple studies. The somas of the CSF-contacting nucleus are located in the brain parenchyma, and the processes extend into the CSF. The anatomical synaptic and non-synaptic relationships between the CSF-CNs and non-CSF-CNs, blood vessels in the brain parenchyma and CSF in the ventricular system have been revealed by the combination of CB-HRP-specific tracing and transmission electron microscope techniques (Zhang et al., 2003; Liang et al., 2007). The results have demonstrated the structural basis for the role of the CSF-contacting nucleus in neurohumoral regulation, providing an explanation not only for why biological substances in the blood and CSF are able to be exchanged in certain physiological and pathological conditions but also for the rapid central effects of drugs or cellular transplants administered via CSF despite the existence of the brain barriers. Some functions have already been applied widely in the clinic. For example, the physical properties, chemical components and cytology in the CSF are detected to diagnose some diseases (Yan et al., 2017). Some drugs that cannot pass through the blood–brain barrier treat diseases more efficiently when administered via the CSF rather than intravenously. Gash and Scott (1980) transplanted hypothalamic tissue into the diacele, which was able to compensate for polydipsia and dieresis caused by the lack of vasopressin (VP) in the Brattleboro rat. By using CB-HRP-specific tracing combined with immunofluorescence double-labeling method, we have identified that many neurons in the CSF-contacting nucleus are serotonergic. The serotonin in the brain is a key neurotransmitter in modulating pain, emotion, stress, and homeostasis, etc. (Hieronymus et al., 2016; Anderberg et al., 2017; Patel and Dickenson, 2018; Grandjean et al., 2019). The serotonergic neurons in the CSF-contacting nucleus may participate in these life activities modulations via neuron–neuron

and neuron–body fluids pathways. Moreover, in previous studies, the distribution and change in the expression of substance P (Lu et al., 2011), acid-sensing ion channel 3 (ASIC_3_) (Wang et al., 2014), transient receptor potential vanniloid-1 (TRPV_1_) (Xu et al., 2011), Wnt_5a_ (Wang J. et al., 2015), extracellular signal-regulated kinase-5 (ERK_5_) (Wang C. G. et al., 2015), mechanistic target of rapamycin (mTOR) and ERK_1/2_ (Li et al., 2015), neurokinin-1 receptor (NK_1_R) (Zhang et al., 2017), and adrenomedullin (Wu et al., 2015), as well as dozens of neuroactive substances (including neurotransmitters, receptors, and ion channel proteins) in the CSF-contacting nucleus in morphine physical dependence (Lu et al., 2011), inflammatory pain (Wang et al., 2014), neuropathic pain (Xu et al., 2011; Li et al., 2015; Wang C. G. et al., 2015; Wang J. et al., 2015), visceral pain (Zhang et al., 2017) and stress (Wu et al., 2015). Studying the characteristics of the CSF-contacting nucleus and understanding the changes in the substances distributed in the nucleus will help elucidate the function of the nucleus and its relationship to physiological processes. In addition, Xing et al. (2015) have reported the role of the CSF-contacting nucleus in sodium sensing and sodium appetite; Fei et al. (2016) have demonstrated the role of the rostroventromedial medulla (RVM) in descending pain regulation originating from the CSF-contacting nucleus; Liu H. et al. (2014) have identified the role of the CSF-contacting nucleus in the descending inhibition of spinal pain transmission. Even so, the true biological functions of the CSF-contacting nucleus remain unclear. Recently, we have used the CB-SAP to specifically damage the CSF-contacting nucleus, and successfully establish the CSF-contacting nucleus “knockout” model animal (Song and Zhang, 2018). The biological functions of the CSF-contacting nucleus can be explored extensively by using this model animal. The somas of the CSF-contacting nucleus make both synaptic (axon terminals connecting with neurons) and non-synaptic (axon terminals connecting with body fluids) connections with non-CSF-CNs, glia cells and blood vessels are located in the brain parenchyma, and the terminals of the processes are able to sense the contents of the CSF and secrete and release into the CSF. Therefore, we speculate that the CSF-contacting nucleus plays a key role in the communication between nerves and body fluids and even immune regulatory systems and may be extensively involved in the regulation of physiological processes.

The significance of this paper is its determination of the pinpoint coordinates of the CSF-contacting nucleus. We anticipate that many scholars will begin to study this intriguing nucleus.

## Author Contributions

L-CZ and S-YS designed the experiments, and captured and made up the figures. All the authors carried out the experiments.

## Conflict of Interest Statement

The authors declare that the research was conducted in the absence of any commercial or financial relationships that could be construed as a potential conflict of interest.
